# 543 Challenges in Burn Nurse and Therapy Staffing During and After a Category 4 Hurricane

**DOI:** 10.1093/jbcr/irac012.171

**Published:** 2022-03-23

**Authors:** Abbey Peterson, Desiree Compton, Dana Y Nakamura, Jeremy Landry, Aimee Keating, Nicole M Kopari, Jeffrey E Carter, Herb A Phelan

**Affiliations:** University Medical Center, Metairie, Louisiana; University Medical Center- New Orleans Burn Unit, New Orleans, Louisiana; University Medical Center- New Orleans Burn Unit, New Orleans, Louisiana; University Medical Center- New Orleans Burn Unit, New Orleans, Louisiana; University Medical Center- New Orleans Burn Unit, New Orleans, Louisiana; Children's Hospital New Orleans, New Orleans, Louisiana; University Medical Center- New Orleans, New Orleans, Louisiana; LSUHSC-New Orleans Department of Surgery, New Orleans, Louisiana

## Abstract

**Introduction:**

Burn nurse/therapy staffing has been stretched for months by the pandemic. Along the Gulf Coast, Hurricane Ida recently taxed these resources further as regional burn centers saw a weeks-long surge in serious burn injuries in the setting of prolonged power and water outages. We reviewed the execution of a plan for the provision of burn nurse/therapist staffing at an ABA-verified adult burn center that experienced a direct hit by a Category 4 storm.

**Methods:**

Hospital leadership planned to activate Code Gray on 8/29/21 at which time the hospital would be placed on lockdown with no one allowed in or out until Code Gray was lifted.

Our burn leadership subsequently designed a plan to have ten burn nurses and one Occupational Therapist (TEAM A) in house from the inception of Code Gray at 7am on 8/29 thru 7am on 9/1. If Code Gray conditions persisted, nine dedicated burn nurses (TEAM B) were to relieve TEAM A. TEAM B was planned to remain in-house until 7am on 9/4. If Code Gray conditions continued, the plan was to be reassessed at that time. The same burn therapist was planned to remain in-house throughout. Physician coverage was to be provided by the in-house trauma team during Code Gray. No housing or bedding was provided for in-house personnel, and the hospital generator system ostensibly had a 30-day fuel supply.

**Results:**

TEAM A day/night staffing was 6/4 with the off crew sleeping in conference rooms and clinic spaces. An unexpected event occurred when a mission-critical tower for the city’s grid toppled into a river resulting in delays for restoration of the grid, and city-wide boil-water and burn-ban policies. As generators came into widespread use, our pre-storm census of 9 increased to a mean of 12.7 + 1.4. Due to this increase, on the morning of 9/1 six TEAM A nurses elected to stay and be absorbed into Team B with day/night staffing of 6/6.

The rapid influx in number and complexity of burn patients made it clear a burn surgeon presence was needed during Code Gray. One burn attending was able to make it to the hospital at 7am on 8/30 and worked until being relieved at 7am on 9/5. An informal triage strategy was enacted in which only burns of >10% TBSA would be considered for admission. OR availability went down to 2 + 1 at the inception of Code Gray and 3 + 1 on 9/6. Eleven cases were done during this time with a mean TBSA of 20.2 + 10.7%.

Hospital generators were found to consume fuel at a rate almost twice predicted. Due to prioritization, the hospital went back on city power on 9/2. Code Gray was lifted at 7am on 9/4 and normal operations resumed at 7am on 9/11.

**Conclusions:**

The successful provision of care required a willingness for nurses and one therapist to remain in the hospital for six consecutive days and for hospital administration to approve the overtime.

